# *Drosophila* taste neurons as an agonist-screening platform for P2X receptors

**DOI:** 10.1038/s41598-020-65169-9

**Published:** 2020-05-19

**Authors:** Leanne Grimes, Julia Griffiths, Gaia Pasqualetto, Andrea Brancale, Paul J. Kemp, Mark T. Young, Wynand van der Goes van Naters

**Affiliations:** 1 School of Biosciences, Cardiff University, Sir Martin Evans Building, Museum Avenue, Cardiff, CF10 3AX UK; 2 School of Pharmacy and Pharmaceutical Sciences, Cardiff University, Redwood Building, King Edward VII Ave, Cardiff, CF10 3NB UK

**Keywords:** Ligand-gated ion channels, Ion channels in the nervous system, Extracellular recording, Receptor pharmacology

## Abstract

The P2X receptor family of ATP-gated cation channels are attractive drug targets for pain and inflammatory disease, but no subtype-selective agonists, and few partially selective agonists have been described to date. As proof-of-concept for the discovery of novel P2X receptor agonists, here we demonstrate the use of *Drosophila* taste neurons heterologously expressing rat P2X2 receptors as a screening platform. We demonstrate that wild-type rat P2X2 expressed in *Drosophila* is fully functional (ATP EC_50_ 8.7 µM), and that screening of small (2 µl) volumes of a library of 80 adenosine nucleotide analogues is rapid and straightforward. We have determined agonist potency and specificity profiles for rat P2X2 receptors; triphosphate-bearing analogues display broad activity, tolerating a number of substitutions, and diphosphate and monophosphate analogues display very little activity. While several ATP analogues gave responses of similar magnitude to ATP, including the previously identified agonists ATPγS and ATPαS, we were also able to identify a novel agonist, the synthetic analogue 2-fluoro-ATP, and to confirm its agonist activity on rat P2X2 receptors expressed in human cells. These data validate our *Drosophila* platform as a useful tool for the analysis of agonist structure-activity relationships, and for the screening and discovery of novel P2X receptor agonists.

## Introduction

P2X receptors are ATP-gated ion channels which play diverse roles in physiology and thereby affect a variety of diseases and conditions, including, although not exhaustively: pain sensation^1^, cancer^2^, arthritis^3,4^, osteoporosis^5^, and hypertension^6^. Together with the metabotropic P2Y receptors, P2X receptors mediate cellular responses to extracellular ATP^7^. ATP functions as an intercellular signaling molecule through release as a (co)transmitter in many synapses, during muscle contraction in skeletal and heart muscle, and by receptor-mediated release in many non-excitable tissues. ATP is also released from cells following damage or stress to the cell membrane by a variety of conductive mechanisms^8^, and is released constitutively at low flux rates^9^. Seven *P2X* genes in mammals (*P2RX1*-*P2RX7*), some of which may be alternatively spliced, encode channel subunits that trimerize to form a complex repertoire of homo- and heterotrimeric P2X receptors. Each subunit has two transmembrane domains connected by a large extracellular loop; N- and C-termini are intracellular. *P2X* genes have also been identified in many other classes of vertebrates and invertebrates, as well as in lower organisms, such as slime mold, where the P2X receptor has an intracellular function in vesicle fusion^10,11^.

The P2X2 subunit in mammals functions as a homotrimer, or in association with other subunits, in signaling in both the peripheral nervous system (PNS) and central nervous system (CNS). In the PNS, P2X2 receptors of the carotid sinus nerve endings are crucial to carotid body oxygen signal transduction^6^. Functions of P2X2 receptors have also been reported in trigeminal and myenteric nerves^12^. In the CNS, P2X2 receptor expression is widespread^7^ with functional receptors identified in cultured Purkinje cells^13^, neurons of the dorsal horn^14^ and neuron terminals in the posterior pituitary^15,16^. In association with P2X3, P2X2 functions in taste sensation, pain and inflammation^17^.

Substantial early research in P2X receptors on ATP binding has been carried out using rat P2X2^18^, and parallel work focused on human P2X1^19–22^. Studies on P2X2 and P2X4 suggested that the mode of binding ATP is common among P2X receptors^23^. The determination of the crystal structures of zebra fish P2X4 in the closed apo-^24,25^ and in the ATP-bound open state^25^, and human P2X3 with bound ATP^26^, has corroborated most of the interactions between the P2X receptor and ATP that were proposed in these earlier studies, in addition to providing detailed insight into the overall architecture and structural rearrangements between the open and closed conformers (reviewed by^27–29^).

**Figure 1:**
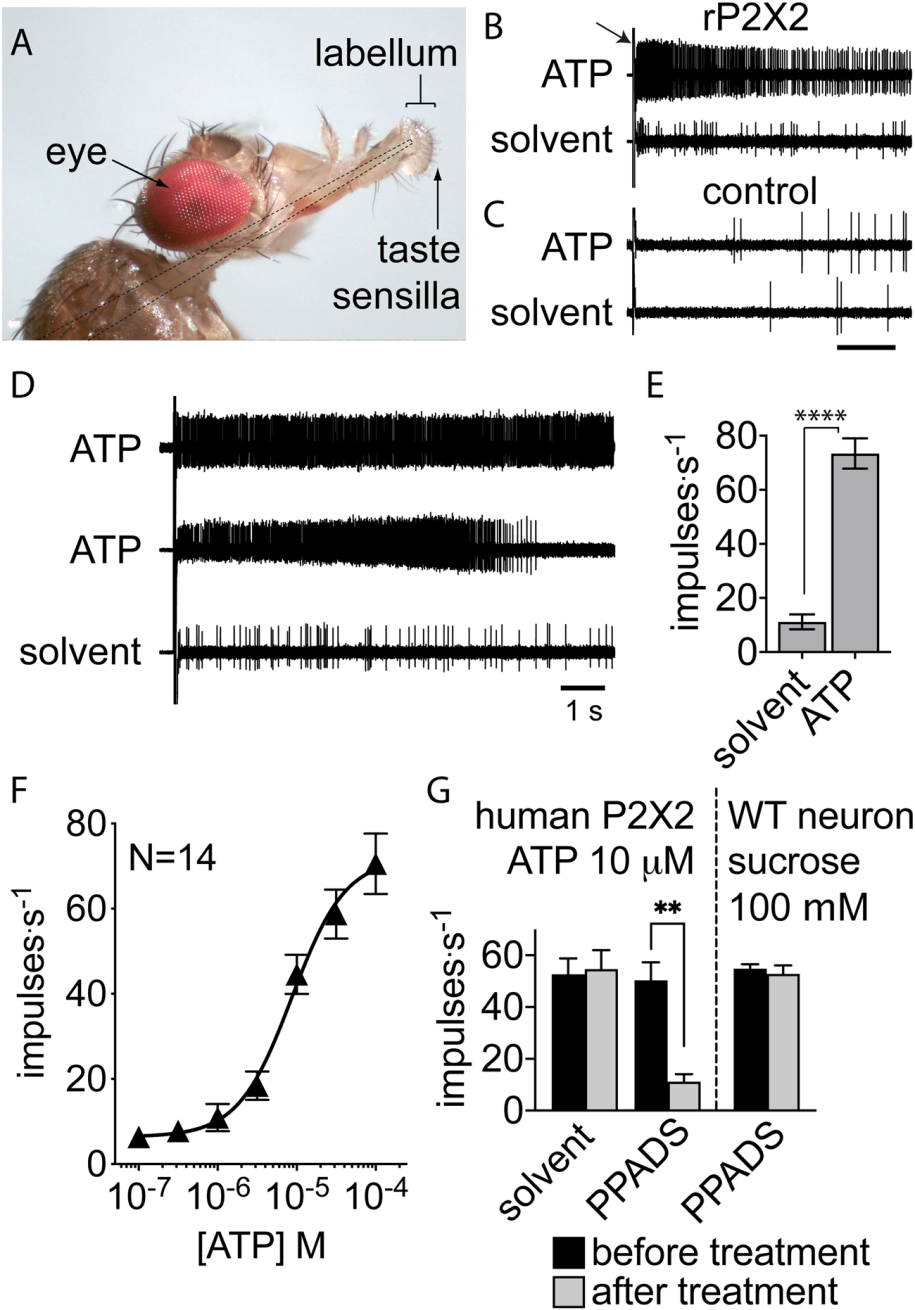
P2X2 mediates responses to ATP in *Drosophila* sugar taste neurons. (**A**) Photo of fly preparation for recording. Dashed lines indicate outline of reference electrode, which is placed in the fly with its tip reaching inside the distal end of the proboscis near the labella. The hair-like taste sensilla are accessible on the exterior with a pipette containing the stimulus. The pipette also serves as recording electrode. (**B**) Recording of action potential response to 100 μM ATP (top) and solvent (bottom) from rat P2X2 expressing flies. Arrow indicates contact artifact when recording pipette makes initial contact with the sensillum. Flies carry a single copy each of *UAS-rat P2X2::mCherry*, where:: denotes a construct encoding a fusion protein, and *Gr5a-GAL4*. (**C**) As in (**B**) but from control flies carrying the *UAS* transgene but no driver to express it. (**D**) Variability in temporal kinetics of responses to ATP in rP2X2 expressing flies: phasic-tonic as in (**B**), tonic (top trace) or with slow onset and rapid off kinetics (middle trace). (**E**) Responses to solvent and 100 μM ATP (mean and SEM, N = 12, P < 0.0001 paired t-test). (**F**) Response to ATP in flies expressing rat P2X2 to increasing concentrations of ATP (mean and SEM, N = 14, EC_50_ = 8.7 μM with 95%CI 5.1 to 14.7 μM). (**G**) Effect of 2 minute treatment of 30 μM PPADS on the subsequent response of human P2X2 to 10 μM ATP (middle pair of bars); responses before and after treatment with PPADS differed significantly (mean and SEM, N = 6, P = 0.004 paired t-test). Solvent treatment had no significant effect on the ATP response in flies expressing P2X2 (shown at left: mean and SEM, N = 6, P > 0.05 paired t-test), and PPADS treatment had no significant effect on the sucrose response in wild-type flies (shown at right: mean and SEM, N = 6, P > 0.05 paired t-test).

The knowledge of the structure of the P2X binding pocket and its interactions with the primary ligand offers a unique opportunity to understand the mechanistic basis of ligand specificity of the receptor. The ligand specificity of P2X receptors has been explored in previous studies (reviewed in^30–32^), and most recently in a systematic study of agonist recognition and specificity in rat P2X2 receptors^33^, where a series of ATP analogues with modifications to the base and ribose were examined. In our study we have further expanded the range of molecules tested on rat P2X2 by using a library of 80 adenosine nucleotides. In order to test this library we developed a rapid and sensitive assay, which uses very small quantities of ligand, based on overexpressing P2X receptors in *Drosophila* taste neurons. Taste neurons in the distal mouthparts of *Drosophila* (Fig. 1A) are housed in hair-like cuticular structures called sensilla^34,35^, which have an apical pore that allows chemicals to enter the sensillum and interact with receptors expressed in the neurons’ dendrites. The architecture of the sensilla, together with their accessible position on the labellae, means that the action potential activity of taste neurons in response to panels of chemicals can be readily recorded. Several adenosine nucleotides gave responses as large as ATP, including the previously identified agonists ATPγS and ATPαS. We also identified a novel agonist, 2-fluoro-ATP (2F-ATP), from our screen in *Drosophila*, and confirmed its activity on rat P2X2 by measuring currents using whole-cell voltage-clamp on HEK cells heterologously expressing the receptor. Rat P2X2 exhibited a relatively broad activity profile to adenosine nucleotides with three phosphoryl groups, but did not respond robustly either to ADP or AMP and their derivatives. This suggested that the binding pocket can accommodate a range of modifications of the primary ligand. A T184I P2X2 receptor mutant showed reduced activity in *Drosophila* taste neurons, which could be explained by loss of hydrogen bonding to ATP as deduced from the crystal structures of other P2X receptors.

In summary, we have developed a sensitive functional assay in *Drosophila* taste neurons for mammalian ligand-gated ion channels, which permits the rapid screening of compound libraries in small (2 µl per application) quantities. We have used this assay to profile the activity of 80 compounds from an adenosine nucleotide library against rat P2X2, discovering a novel agonist and expanding our understanding of the structure-activity relationship of agonist action.

## Results

### Rat P2X2 is functional in taste neurons of *Drosophila*

To determine whether rat P2X2 formed a functional channel in *Drosophila* taste neurons, we tested the responses to ATP in taste neurons of flies expressing rat P2X2 via the *GAL4-UAS* system under control of the *Gr5a* promoter. ATP at 100 μM elicited strong excitatory responses from taste neurons in sensilla on the mouthparts of these flies, while the solvent by itself did not (Fig. 1B), suggesting rat P2X2 is functional in the sugar taste neuron. Moreover, *Drosophila* taste neurons do not show endogenous responses to ATP; control flies carrying a transgene of *UAS-ratP2X2* but without a *GAL4* driver did not respond to ATP (Fig. 1C). We noted that in solvent-only recordings, flies expressing rat P2X2 exhibited higher action potential activity than control flies (Fig. 1B and C). The temporal kinetics of the responses to ATP in flies expressing rat P2X2 varied from phasic-tonic (Fig. 1B), to tonic (Fig. 1D, top trace) while many neurons showed a delayed onset of response (Fig. 1D, middle trace). The response to ATP differed significantly from the solvent response (Fig. 1E). The response to ATP mediated by rat P2X2 was concentration-dependent (Fig. 1F), with an EC_50_ of 8.7 μM (95%CI: 5.1 to 14.7 μM). The human P2X2 ortholog was also tested in *Drosophila* taste neurons, as we intend to study the human receptor after beginning with the rat receptor as a proof-of-concept, and it responded to ATP in a similar fashion. Among several antagonists of P2X receptors that have been identified, PPADS acts strongly on homomeric P2X1, P2X2, P2X3 and P2X5 receptors (reviewed in^30^). To test if P2X2 is similarly inhibited in the context of the *Drosophila* taste system, we applied it for two minutes at 30 μM and observed strong inhibition of the ATP response (Fig. 1G). A two-minute solvent treatment did not affect the ATP response. In addition, the sucrose response in wild-type flies was not significantly affected by PPADS treatment, which suggests that the inhibitor indeed acted directly on the expressed P2X receptor.

### Screening adenosine nucleotides validates known ligands and identifies additional agonists

Having determined that rat P2X2 forms a functional channel in taste neurons of *Drosophila*, we proceeded to screen a library of 80 adenosine nucleotides for their agonist activity. A small number of chemicals in the library have been previously tested on P2X receptors, as described below, and were useful in validation of the *Drosophila* assay platform.

**Figure 2:**
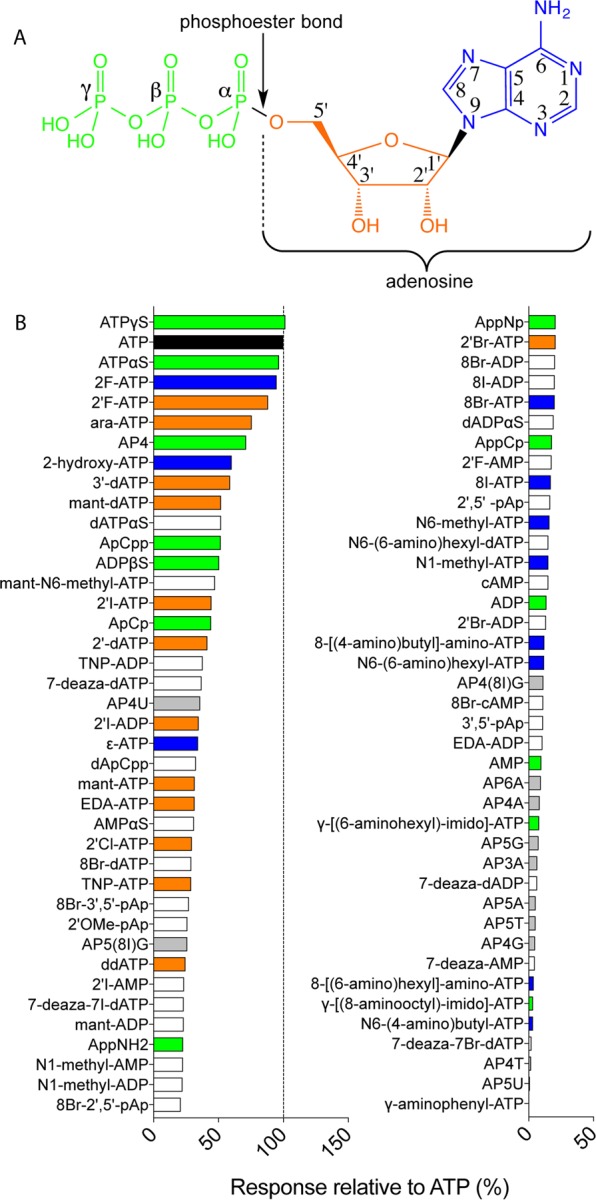
Responses in taste neurons expressing rat P2X2 to a library of adenosine nucleotides. (**A**) Structure of ATP. The adenine base (in blue) is linked to C1′ of the sugar ribose (in orange) to which three phosphoryl groups (in green) are connected at C5′. (**B**) Mean response to 80 adenosine nucleotides tested at 100 μM, N = 3–6. Nucleotides are arranged in order of activity relative to the response to ATP (in black). Derivatives of ATP include modifications in the phosphoryl groups (green), the ribose moiety (orange), the adenine base (blue), a combination of two or more of these (white) or are dinucleotides (grey).

In ATP the adenine base is linked to C1′ of the sugar ribose to which three phosphoryl groups are connected at C5′ (Fig. 2A). The library comprised chemicals with changes in the adenine base, the ribose sugar, and the phosphate moieties of ATP and combinations of changes in these three moieties, as well as a number of dinucleotides. Responses to the 80 nucleotides ranged in a smooth progression from full agonist activity, with responses similar to ATP, to no response (Fig. 2B). We did not find nucleotides that exceeded the activity of ATP on the receptor by >20%. For all three moieties, there were some changes that had little effect on agonist activity: in ATPγS the gamma phosphoryl group is replaced with a thiophosphoryl group, 2F-ATP has a fluoro substitution on the adenine base, and 2′F-ATP has a fluoro substitution on the ribose ring. ATPγS and ATPαS have previously been shown to act as agonists on P2X2 expressed in *Xenopus* oocytes^36^ and both were also effective agonists in the *Drosophila* system. 2F-ATP, ATPγS and ATPαS did not elicit responses greater than that of solvent alone in control flies not expressing rat P2X2 receptors (data not shown).

The presence of three phosphoryl groups is a necessary condition for strong agonist activity, as none of the ADP and AMP derivatives elicited >50% of the response to ATP. However, it is not a sufficient condition, as most modifications of chemicals with three phosphoryl groups still led to a reduction or complete loss of activity at 100 μM. Additions to, or substitutions in, positions 1, N6, 7 and 8 of the adenine ring were all detrimental, while position 2 allowed some modification (2F-ATP and 2OH-ATP). In the ribose ring, changes were detrimental to agonist activity both at C2′ (but 2′F-ATP showed some activity) and C3′.

We noted that diadenosine tetraphosphate (AP4A) did not elicit responses from rat P2X2 in the *Drosophila* taste system, although it has been reported to be a full agonist when expressed in *Xenopus* oocytes^37^.

### Whole-cell voltage clamp of rat P2X2 in HEK cells validates a novel agonist

**Figure 3:**
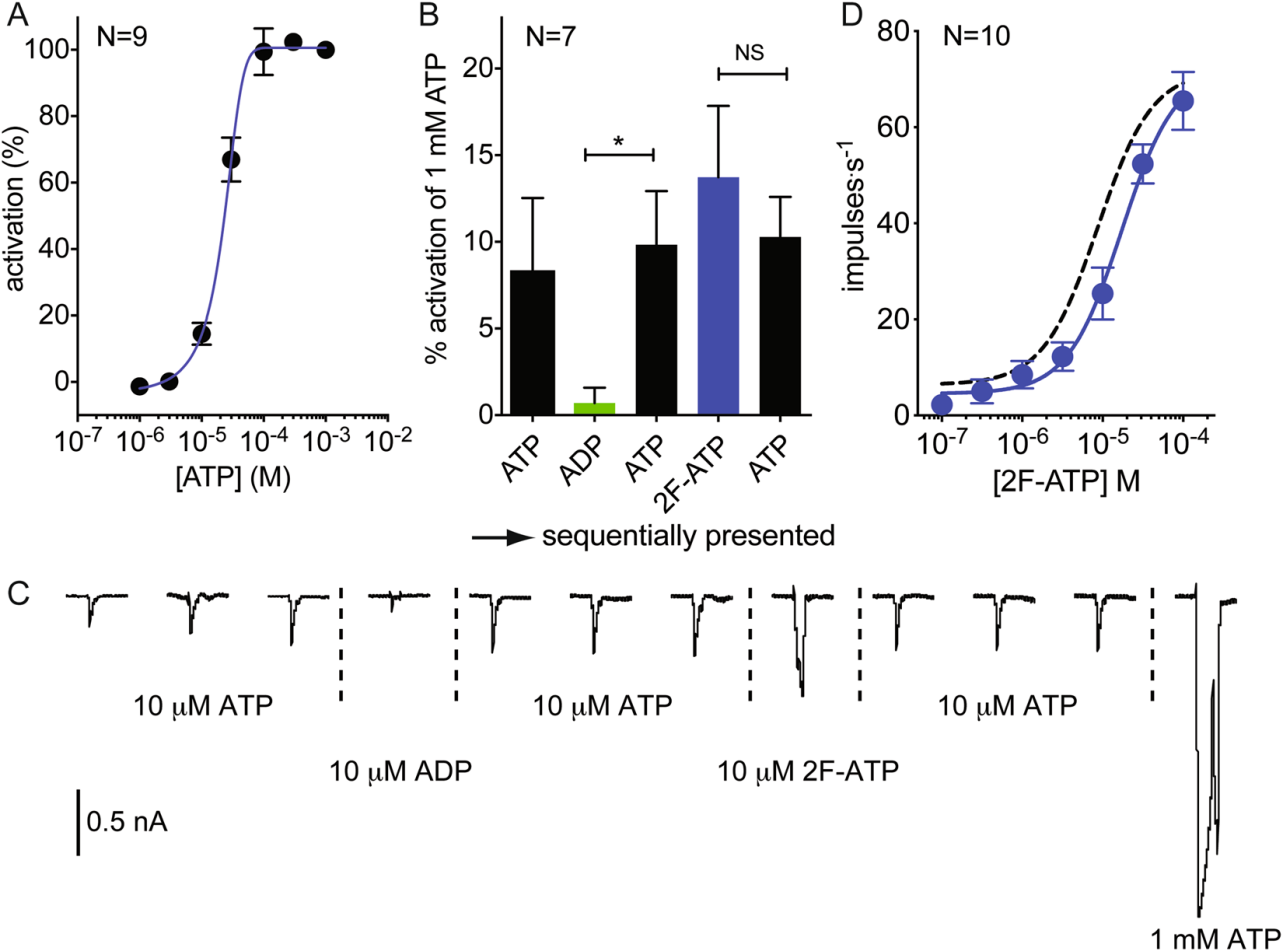
Responses mediated by rat P2X2 confirm 2-fluoro-ATP (2F-ATP) is a full agonist. (**A**) Recordings under whole-cell voltage clamp from HEK cells expressing rat P2X2 show the response to ATP increases with concentration. (**B**) Responses to ATP, ADP, ATP (repeat stimulus), 2F-ATP and ATP (repeat stimulus) presented sequentially each at 10 μM, relative to ATP at 1 mM (mean and SEM, *P < 0.05 ANOVA with Bonferroni post-test, NS non-significant). (**C**) Current traces in one of the experiments underlying data in (**B**). Applications of ADP, 2F-ATP and 1 mM ATP each followed three applications of ATP. (**D**) Response to 2F-ATP in flies expressing rat P2X2 to increasing concentrations (mean and SEM, N = 10, EC_50_ = 17.2 μM with 95%CI 9.5 to 31.3 μM). Black dashed curve is fitted to ATP data from Fig. 1F for comparison.

Screening the library on rat P2X2 expressed in *Drosophila* taste neurons led to the identification of 2-fluoro-ATP (2F-ATP) as a novel full agonist. To confirm its activity in an established expression platform, 2F-ATP was presented to HEK 293 cells stably expressing rat P2X2 receptors. We first verified the responsiveness to ATP of these cells by measuring whole-cell transmembrane currents in voltage-clamp to concentrations from 10^–6^ to 10^–3^ M; ATP elicited concentration-dependent cationic currents in this range (Fig. 3A). Subsequently, responses were recorded to ATP, ADP and 2F-ATP at 10 μM. 2F-ATP elicited currents that did not differ significantly from currents elicited by ATP (Fig. 3B,C), which provided an independent experimental validation of the finding from the *Drosophila* assay. Due to the high cost of 2F-ATP, we were unable to purchase sufficient volumes to generate a concentration response curve using voltage-clamp in HEK 293 cells. However, this was possible in the *Drosophila* system due to the small application volumes required (2 µl). We found that the potency of 2F-ATP was similar to that of ATP, with EC_50_ values of 17.2 μM (Figs. 3D) and 8.7 µM (Fig. 1F), respectively.

### An amino acid substitution in the ATP binding pocket alters ligand activity in P2X2

**Figure 4:**
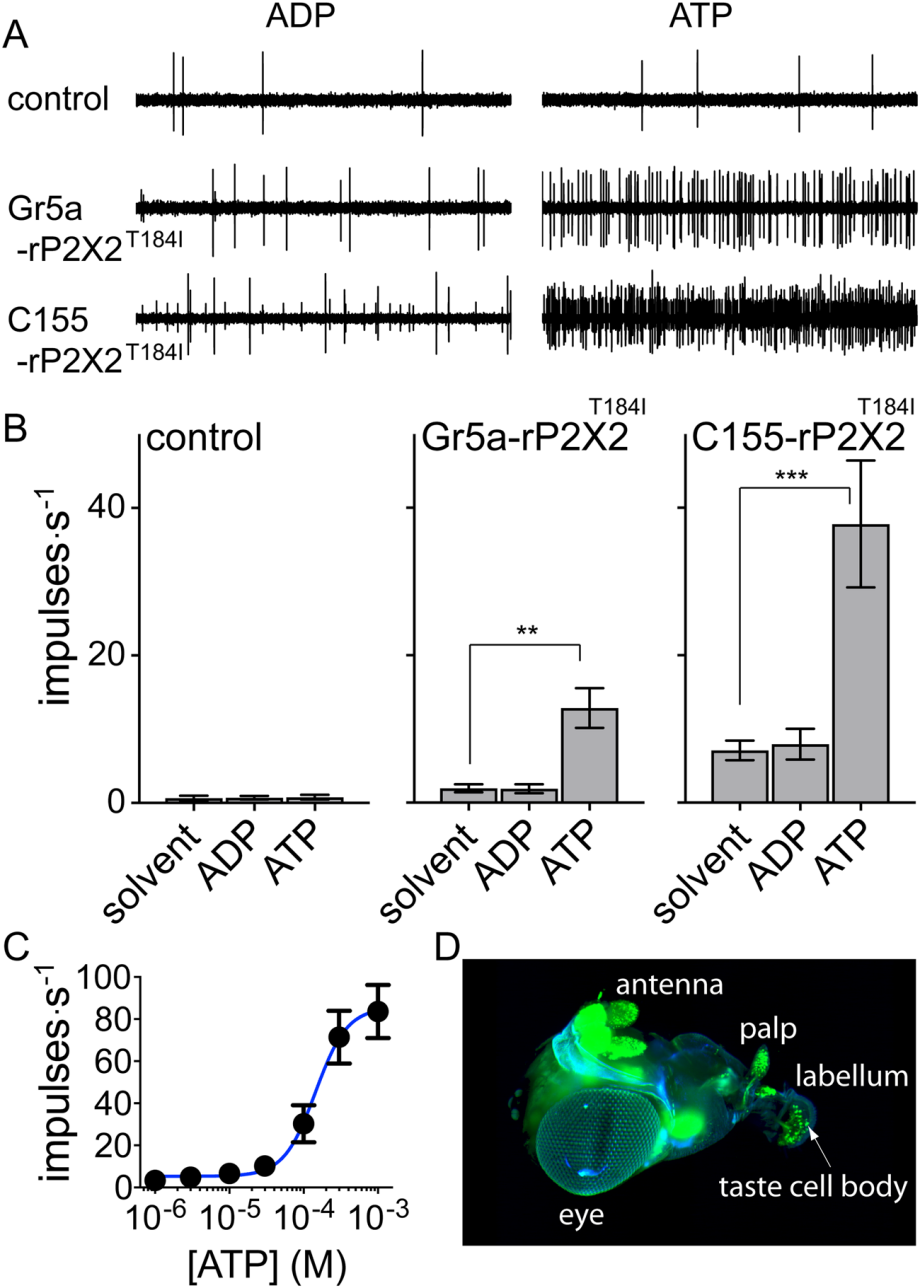
Responses mediated by T184I rP2X2::GFP in taste neurons. (**A**) 100 μM ATP elicits responses from mutated rP2X2 while 100 μM ADP does not. Top traces from control flies carrying a UAS transgene but no driver, middle traces from flies expressing T184I rP2X2 in sugar taste neurons under control of the *Gr5a* promoter, bottom traces from flies with pan-neural expression of T184I rP2X2 under control of the *C155* driver. (**B**) Mean responses (with SEM) to solvent, ADP and ATP in control flies and in flies expressing T184I rP2X2 in sugar taste neurons and in all neurons (ns p ≥ 0.05 **p < 0.01 ***p < 0.001 paired Anova and post-hoc Dunnett’s test). Solvent elicited impulse rates among genotypes differ significantly between *Gr5a-rP2X2*^*T184I*^ and *C155-rP2X2*^*T184I*^, p = 0.0001 (unpaired Anova and post-hoc Dunnett’s test). (**C**) Response to ATP in flies expressing rat P2X2 to increasing concentrations of ATP (mean and SEM, N = 14, EC_50_ = 145 μM with 95%CI 92 to 370 μM). (**D**) Light-sheet fluorescence micrograph of neurons with pan-neural expression of T184I rP2X2::GFP (in green).

We serendipitously generated a *Drosophila* line of rat *P2X2* encoding the channel with a T184I substitution. Thr-184 is a conserved residue that corresponds to T189 in zebra fish P2X4 and to T172 in human P2X3. This residue has been shown in structural studies to interact through hydrogen bonding with the adenine moiety of ATP in the P2X binding pocket^25,26^, and substitution of this residue has previously been demonstrated to reduce ATP potency and activity^18^. The sugar taste neurons expressing the T184I mutant still responded to ATP at 100 μM, albeit with slow onset kinetics and low impulse rates (Fig. 4A,B). To broaden and enhance expression in the other taste neurons of the sensilla, flies carrying either WT or T184I *UAS-rat P2X2* were crossed to a strong pan-neural driver. Preliminary observation of flies with pan-neural expression of WT rat P2X2 suggested that the ion channel interferes with neuronal physiology. The flies exhibited a tremor or shaking phenotype at rest and their locomotor activity appeared to be inhibited. These phenotypes appeared three days post-eclosion in flies expressing the WT rat P2X2 but were absent in flies expressing the T184I variant. Testing concentrations from 10^–6^ to 10^–3^ M showed that ATP was less potent at the T184I receptor (Fig. 4C), with an EC_50_ of 145 μM (95%CI: 92 to 370 μM), than at the WT receptor (Fig. 1F). Microscopy showed strong expression of the T184I-GFP fusion protein under control of the pan-neural driver in neurons throughout the animal (Fig. 4D), suggesting low responses to ATP were not due to low levels of expression or poor dendritic localization. Also, while it is possible that the low responses to ATP were caused by interference of the C-terminal GFP fusion tag with the function of the receptor, previous studies on rat P2X2 expressed in *Xenopus* oocytes showed that responses mediated by a C-terminal GFP fusion tagged receptor and a wild-type receptor are similar in EC50, peak current and desensitization kinetics^38,39^. Rat P2X3 constructs carrying other C-terminal fluorescent tags (ECFP or DsRed2) also did not differ from wild-type receptors in their affinity for the ligand or in their ion conductance properties^40^, suggesting that members of the P2X family may generally be tolerant of C-terminal fluorescent protein tags.

**Figure 5:**
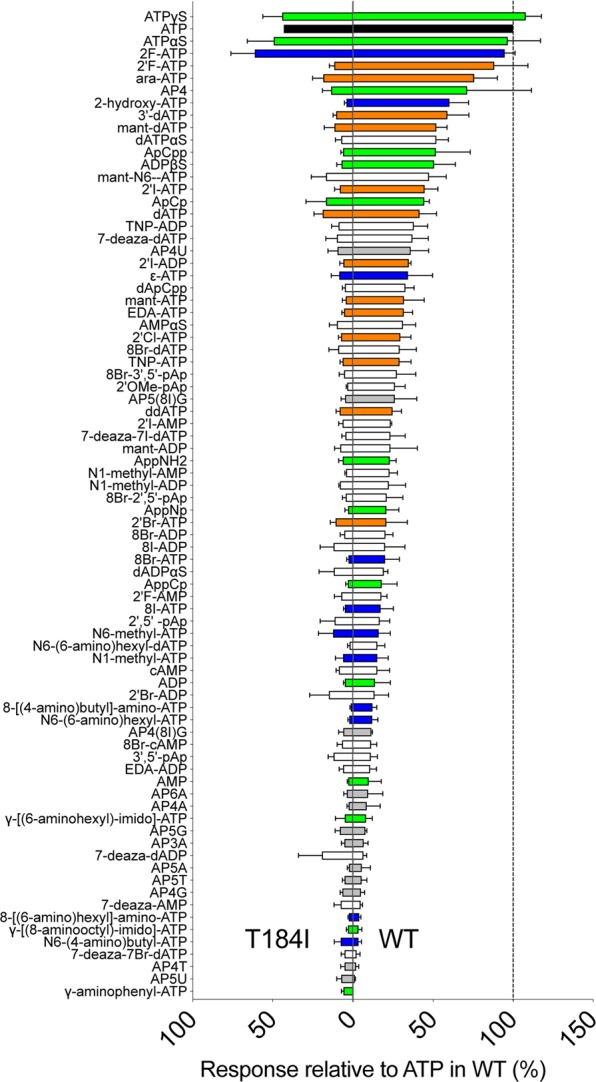
Comparative response to 80 adenosine nucleotides tested at 100 μM at wild-type and T184I rat P2X2 (mean and SEM, N = 3–6) expressed in *Drosophila* taste neurons. Wild type (WT) rat P2X2 (data from Fig. 2) at right and T184I mutant at left. Nucleotides are arranged in order of activity relative to the response to ATP in WT. ATP (black) and its derivatives include modifications in the phosphoryl groups (green), the ribose moiety (orange), the adenine base (blue), a combination of two or more of these (white) or are dinucleotides (grey).

We subsequently tested the library of 80 adenosine nucleotides on flies expressing rat P2X2^T184I^ and found that ATPγS, ATPαS and 2F-ATP elicited responses similar to ATP (Fig. 5); mirroring the situation in the WT receptor. However, the ribose modified 2′F-ATP, ara-ATP and the nucleotide with four phosphoryl groups AP4 as well as 2OH-ATP elicited no significant responses even though the WT rat P2X2 mediated an intermediate response to these ligands (see Fig. 2).

**Figure 6:**
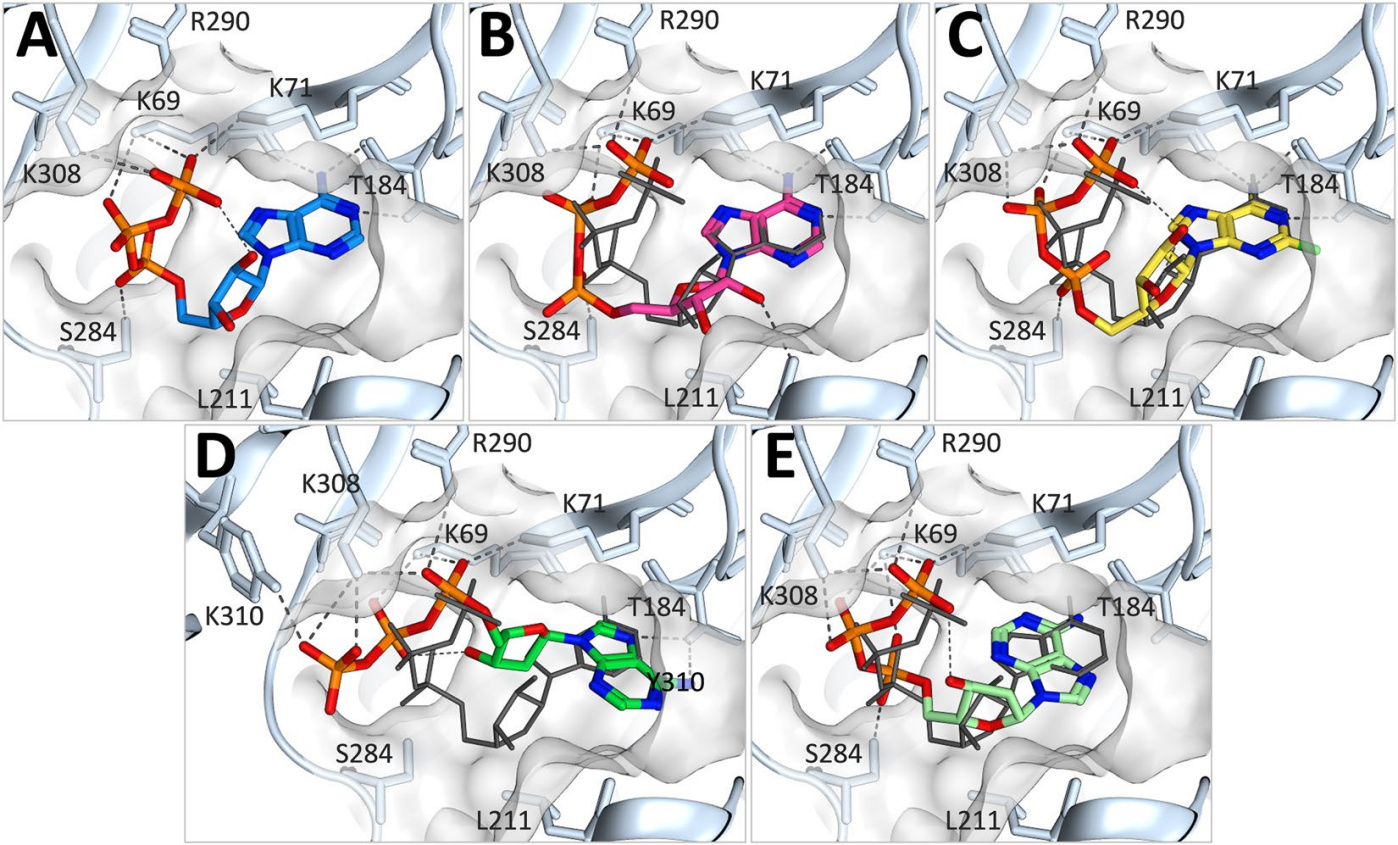
Docking of ATP, 2F-ATP and 2′-dATP into the ATP binding site of rat P2X2. (**A**) Molecular model of rat P2X2 derived from the ATP-bound crystal structure of zebrafish P2X4 (PDB ID 4DW1) showing the key polar contacts (dashed lines) involved in ATP binding. (**B**) Representative pose from molecular docking of ATP into the rat P2X2 model using Glide XP (pink and orange sticks), compared to ATP from the molecular model (dark grey lines; RMSD = 1.4799). The position of the adenine ring is near-identical and the overall conformation, positioning and interactions of the phosphates are very similar. The ribose moiety is rotated, and the 2′ hydroxyl forms a hydrogen bond with the main chain of Leu-211. (**C**) Docking of 2F-ATP (yellow and orange sticks) compared to ATP from the molecular model (RMSD = 1.1970). In the 2F-ATP dock, the hydrogen bond between the 2′ hydroxyl and the γ-phosphate oxygen is maintained. (**D**,**E**) are representative poses obtained for 2′-dATP; either the U-shaped conformation of ATP is not maintained (**D**); RMSD = 4.0540), or the adenine ring is in a very different orientation (**E**), probably induced by a change in conformation of the ribose to accommodate a hydrogen bond between the 3′ hydroxyl and the γ-phosphate oxygen. N288 is not labeled for clarity.

In order to rationalize our experimental data, we constructed a homology model of rat P2X2 based on the zebrafish P2X4 ATP-bound crystal structure^25^. In the modeling procedure, the conformation of ATP was constrained, and so matched that observed in the original crystal structure. The ATP binding site, indicating the key conserved amino acid residues which contribute polar contacts, is shown in Fig. 6A. The side-chain hydroxyl group of Thr-184 forms a hydrogen bond with the N1 position of the adenine ring, which would be disrupted in the T184I mutant, explaining the lower potency and activity of ATP at this mutant receptor. In order to assess the binding of other nucleotide analogues to our model, we first performed molecular docking simulations with ATP (Fig. 6B). The docked ATP displayed very similar conformation to that of the ATP derived from the crystal structure (RMSD = 1.4799), with one notable difference: instead of forming an intramolecular hydrogen bond with the γ-phosphate oxygen, the 2′ hydroxyl group of the ribose formed an intermolecular hydrogen bond with the main chain of Leu-211. Docking with the novel full agonist 2F-ATP (Fig. 6C) predicted a docking conformation more similar to that of ATP (RMSD = 1.1970), preserving the intramolecular hydrogen bond and demonstrating that the fluorine substitution is well accommodated in the binding pocket. It has recently been demonstrated that 2′-dATP is not an effective agonist at P2X1 receptors. This has been proposed to arise from the loss of an intramolecular hydrogen bond between the 2′ hydroxyl and a γ-phosphate oxygen which destabilizes the U-shaped conformation of ATP required for efficient agonist action^41^. In our molecular docking simulations using 2′-dATP (Fig. 6D,E), we observed two distinct conformations of 2′-dATP; one where the U-shaped conformation was lost, leading to a significant translation of the adenine ring and complete rearrangement of the critical lysine-phosphate interactions (Fig. 6D, RMSD = 4.0540), and one where the U-shaped conformation was maintained by formation of an intramolecular hydrogen bond between the γ-phosphate oxygen and the 3′-hydroxyl of the ribose (Fig. 6E) leading to a significant translation of the adenine ring relative to that observed in our molecular model derived from the ATP-bound crystal structure.

## Discussion

Heterologously expressing a mammalian P2X receptor in *Drosophila* taste neurons allowed us to rapidly screen 80 adenosine nucleotides for agonist activity, suggesting that P2X receptors can function alongside insect taste receptors in mediating action potential responses to ligands in taste neurons. Sugar taste in *Drosophila* is mediated by receptors encoded by members of the *Gr* gene family^42,43^, but the mechanism by which sugar reception is transduced to an action potential response is not yet clear. The sugar receptor BmGr-9 from the silkworm *Bombyx mori* mediates responses to D-fructose when expressed in *Xenopus* oocytes or HEK 293 cells^44^. Pharmacological experiments suggest that Ca^2+^ influx in response to fructose does not depend on G-protein coupled cascades, and further electrophysiological analyses of macroscopic and unitary currents supported the hypothesis that BmGr-9 forms a non-selective cation channel. P2X receptors are ligand-gated ion channels permeable to cations including K^+^, Na^+^ and Ca^2+^, although P2X5 also carries Cl^−^^45^. In rat P2X2, the Ca^2+^ current contributes 5.7% of the whole cell current, as measured by patch-clamp photometry^46^. Therefore, activation of P2X2 receptors can lead to membrane depolarization and may also affect cellular physiology through Ca^2+^ influx. The analogies in function between mammalian P2X receptors and an insect sugar Gr suggest sugar taste neurons may provide a useful and responsive expression system to assay P2X ligand specificities. The EC_50_ of 8.7 μM for rat P2X2-mCherry expressed in sugar taste neurons is indeed similar to the potency of ATP for rat P2X2 and rat P2X2-GFP expressed in *Xenopus* oocytes (EC_50_ = 6.5 µM and 13.5 µM, respectively^38^), and HEK 293 cells^47^. Rat P2X2 receptors have been previously expressed in *Drosophila* neurons and were shown to be functional in the CNS^48^ using both bath application of ATP on cholinergic neurons expressing the receptor and photo-release of caged ATP for the receptor expressed in the Giant Fiber system. Our experiments show that rat P2X2 is also functional in the peripheral nervous system.

As an assay platform, the *Drosophila* taste system is a neuronal expression system with endogenous receptors whose mechanisms of transduction show some analogy to P2X receptors, as discussed above. It may therefore be particularly suitable for functional studies of neuronally expressed P2X2/P2X3 heteromeric- or P2X3 homomeric receptors that are thought to act in nociceptive, inflammatory and neuropathic pain^49,50^. Some further practical considerations support the value of the *Drosophila* taste system as an assay platform for P2X receptors: (1) stimuli can be prepared in small quantities as a presentation can be achieved using only 2 μl of solution; (2) once transgenic flies are obtained, maintenance of the flies is straightforward and economical; (3) taste recording from insect sensilla is a well-established technique^51^. However, the observation that flies with pan-neural expression of wild-type rat P2X2 showed behavioral phenotypes three days after eclosion suggests that the receptor interferes to some extent with neuronal physiology in adults. Strong pan-neural expression of rat P2X2 under control of the *elav-GAL4* driver line was previously reported to cause subtle incoordination in *Drosophila* while lower expression levels under control of a weaker driver line did not^48^. Larvae of *Drosophila* with pan-neural expression of rat P2X2 did not show conspicuous phenotypes. While members of the P2X receptor family are present in diverse clades of the Metazoa, they appear to be absent in insects^52^ and our observations suggest adult *Drosophila* may be affected by expression of ligand-gated channels with high sensitivity for extracellular ATP. We do not know whether the extracellular space around *Drosophila* neurons contains enough ATP to activate P2X channels, but taste neurons expressing rat P2X2 did show a higher baseline action potential activity than neurons in control flies even in the absence of experimentally applied ATP. A larger frequency of miniature excitatory junction potentials in recorded muscles was also noted when rat P2X2 was expressed in cholinergic neurons innervating abdominal muscles via the glutamatergic motor neurons^48^. Single channel recordings on P2X2 have shown no spontaneous channel opening^53,54^, so the increased baseline of activity that we observed is unlikely to be caused by stochastic opening of non-stimulated channels. Since the expression of the T184I mutant did not appear to affect the behavior of the flies, poor tolerance for high levels of mammalian membrane protein expression *per se* do not seem to underlie the behavioral phenotypes observed in flies expressing WT rat P2X2 receptors.

Here, we have demonstrated that 2F-ATP is a novel full agonist for rat P2X2, and have shown computationally that it adopts a very similar conformation to ATP in the P2X2 receptor binding pocket. Together with partial activity of 2-hydroxy-ATP tested here, and full activity of 2-chloro-ATP reported previously^55^, we suggest that some substitutions on the 2-position of the adenine base are well-tolerated. Diadenosine tetraphosphate (AP4A) did not elicit responses in the taste neurons although it was reported to act as a full agonist at 100 μM in *Xenopus* oocytes^37^. In this regard, it might be informative to examine the activity of AP4A on P2X2 receptors expressed in other systems, such as HEK 293 cells. However, the taste neuron responses to ATPγS and ATPαS, the conserved low responses to αβ-meATP (ApCpp) and βγ-meATP (AppCp) reviewed in^30,32^, together with our direct demonstration of reduced ATP potency and activity of the T184I mutation^18^, would suggest very strongly that rat P2X2 in the *Drosophila* system does function similarly as it does in other heterologous systems. This means the variance we have demonstrated in activity of AP4A at the rat P2X2 receptor are likely to be due to differences in the expression systems used.

The compound library comprises a variety of adenosine nucleotides and most show less activity than ATP, indicating that several different moieties of ATP can be recognized in the ATP binding pocket. Indeed, earlier findings from systematic alanine and cysteine scanning mutational analyses have pointed to an extensive number of residues that co-ordinate binding of ATP (reviewed by^56^) and this is corroborated by the solved P2X structures. Lysine residues interact with the phosphoryl groups (in rat P2X2 numbering: K69, K71, and K308 through hydrogen bonds and K188 via solvent molecules) as do N288 and R290. The ribose ring is recognized through hydrophobic interactions with L211 and the adenine moiety is co-ordinated by hydrogen bonding with K69 and T184 as well as through hydrophobic interactions with L186 and I226. T184 interacts with the N atoms in adenine at N1 and C6 through two hydrogen bonds. Indeed, substitution of this residue greatly reduced the potency and activity of ATP in our study, as has been shown previously^18^. Adenine position C2 does not appear to be co-ordinated by the binding pocket, and several modifications at this position show full (2F-ATP) or partial (2-hydroxy-ATP) agonist activity. In contrast, several chemicals, with modifications of ATP at positions that do not interact with residues in the binding pocket in the solved P2X structure, such as in 2′-dATP or modifications at adenine C8, did display reduced activity. Fryatt *et al*. reported that while 3′-dATP was a full agonist at P2X1 receptors, 2′-dATP displayed no agonist activity^41^. They used molecular dynamics simulations to demonstrate that the loss of an intramolecular hydrogen bond between the 2′ hydroxyl and a γ-phosphate oxygen could destabilize the U-shaped conformation of ATP required for efficient agonist action. In terms of rat P2X2 receptors, Gasparri *et al*. found that for 3′-dATP the EC_50_ was increased by a factor of 3, but that 2′-dATP displayed very little agonist activity^33^. Similarly in our study, both 3′- and 2′-dATP displayed reduced activity compared to ATP, with 2′-dATP having the lowest activity, suggesting broad agreement between the agonist profiles of P2X1 and P2X2. However, in our docking simulations, we observed two 2′-dATP binding modes to rat P2X2; one where the U-shaped conformation was lost, and one where maintenance of the U-shaped conformation required an intramolecular hydrogen bond between the γ-phosphate oxygen and the 3′-hydroxyl of the ribose, which induced a significant translation in the adenine ring. One intriguing possibility which emerges from our docking simulations is that ATP may adopt different conformations within the binding pocket. For example, our docking simulations with ATP never showed the requirement of the intramolecular hydrogen bond, instead, the 2′-hydroxyl of the ribose formed an intermolecular hydrogen bond with the main chain of Leu-211; this was observed in multiple poses. However, in our docking simulations with 2F-ATP, we observed the intramolecular bond in every conformation where the adenine ring and the triphosphate positions were preserved. While it seems critical for agonist action that the phosphates (particularly the γ-phosphate) and adenine ring are in the correct conformation, it may be possible that the ribose can be stabilized in different conformations in the binding pocket either by intermolecular or intramolecular hydrogen bonds. In either situation, loss of this hydrogen bond (by loss of the 2′-hydroxyl group) would have a negative effect on the stability of the agonist in the binding pocket. An alternative explanation, offered by Gasparri *et al*., based upon their observations that both 2′-F-dATP and the C3′-endo-locked LNA-ATP displayed significant agonist activity, is that it is the sugar pucker conformation of the ribose, rather than the propensity for intramolecular hydrogen-bonding, that is important for agonist recognition^33^, and our docking simulations do not discount this.

In our study, rat P2X2 exhibited a relatively broad response profile among the 80 adenosine nucleotides, which ranged smoothly from no response to full agonist activity. The solved crystal structures show that ATP is bound at the extracellular loop interface between two subunits within a trimer^25,26,57,58^, as was also suggested by previous research^59,60^. While the holoprotein can thereby bind three ATP molecules simultaneously, only two functional binding sites are required to be occupied in order to facilitate channel opening^61^. Careful early work on single channel opening kinetics had already suggested multiple binding steps and co-operative binding of the three ATP molecules to P2X2^53^. It is now apparent from the work of Browne and North^62^ that co-operativity extends to nucleotides other than ATP. Thus, nucleotides such as CTP, UTP and even ADP are able to affect channel opening in the presence of very low concentrations of ATP, as inferred from whole-cell current responses, suggesting that binding of one molecule of ATP results in an intermediate closed channel state that permits further binding and activation of the channel by other nucleotides^41^. This result implies that the P2X2 binding pocket can accommodate diverse nucleotides and that these nucleotides can contribute to channel opening. Indeed, the sensitization of P2X receptors by a priming ATP stimulus has recently been used to probe the binding affinity of P2X for other nucleotides^63^. Therefore, one interpretation of our results is that the adenosine nucleotides were applied against a low background of ATP present in the extracellular space, as discussed above, so that we were able to obtain responses from a broader range of nucleotides than we would get from a naïve receptor. However, for the range of adenosine nucleotides tested here, there is no comparable study in the literature, so it is rather difficult to assess the extent to which the ligand specificity of rat P2X2 in *Drosophila* recapitulates its function in other heterologous systems. It is intriguing to speculate that a receptor such as P2X2 may have more than one palette of ligands to which it responds when its binding sites are successively occupied.

## Methods

### *Drosophila* stocks

Transgenic constructs were injected into embryos of a phiC31 expressing *attp40* stock (stock 13–20 from the Cambridge Fly Facility) to obtain inserts in a 2^nd^ chromosome landing site. Transgenic flies were crossed to flies carrying *Gr5a-GAL4* (a gift from Anupama Dahanukar) and to flies of C155, which is an enhancer trap line expressing *GAL4* under control of the *elav* promoter^64^, to obtain flies that drive expression of rat *P2X2* under control of the *Gr5a* promoter in the sugar cell of the *Drosophila* taste sensilla (*w*; *UAS-ratP2X2*; *Gr5a-GAL4*) or pan-neurally (*elav-GAL4*; *UAS-ratP2X2*). Adult flies having pan-neural expression of rat P2X2 developed a shaking behavioral phenotype 3–5 days after eclosion, so flies were used for electrophysiology on the day after eclosion only. Adult flies expressing a mutant copy of rat P2X2, which causes an amino acid substitution in the predicted ATP binding pocket of the channel (T184I: see below), did not exhibit such a phenotype and were used 5–10 days after eclosion. As a wild-type stock, *Oregon-R* was used.

### Construction of transgenes

The coding sequence of rat P2X2 in pcDNA3.1 (clone p698, a gift from Alan North) was amplified by PCR and ligated upstream and in-frame with *mCherry* in a pUAST-attB(x19) vector, which is the pUAST-attB vector modified to contain 19 UAS sites in tandem for enhanced expression (gift of Sonia Lopez de Quinto, Cardiff University, U.K.). The coding region for human P2X2 variant A (also called variant 1: NCBI reference sequence NM_170682.4) was obtained by synthesis (Life Technologies) and ligated in the pUAST-attB(x19) vector with *mCherry* to generate a construct encoding human P2X2 with a C-terminal mCherry fusion tag. All constructs were verified by sequencing. In addition to a construct with the wild type (WT) rat *P2X2* sequence, a mutant clone encoding T184I in the rat *P2X2* sequence was generated serendipitously. This mutant was ligated in place of zebra fish *P2X4* in the zfP2X4.1 construct (kindly provided by Eric Gouaux), whereupon the rat *P2X2-GFP-His tag* sequence was amplified by PCR and ligated into pUAST-attB(x19).

### Electrophysiology

Flies were prepared for electrophysiology (Fig. 1A) as described in Benton and Dahanukar^65^. Chemical stimuli were presented and action potentials were recorded from gustatory neurons using standard methods and apparatus, as described previously^43^. Briefly, the open tip (<20 μm outer diameter) of a recording electrode containing the stimulus solution was placed over the distal end of an L3,4 or L7–9 labellar taste sensillum (nomenclature following^66^) so that action potentials from the neurons were recorded extracellularly as the stimulus was presented. Stimulus solutions were prepared by diluting 1 mM stock solutions of chemicals from a library of 80 adenosine nucleotides (Jena Biosciences) to 100 μM in water containing 30 mM tricholine citrate. Tricholine citrate in the solvent provides conductance and suppresses activity of the water neuron, but has no appreciable effect on the other taste neurons^67^. Stock solutions were stored frozen at −20°C, and the 100 μM solutions were prepared daily; during experiments they were kept cold on crushed ice until required. PPADS (pyridoxal phosphate-6-azo(benzene-2,4-disulfonic acid) tetrasodium salt was obtained from Sigma-Aldrich. Recordings were band-pass filtered between 50 Hz and 5 KHz and digitized at 10 KHz. Basic analysis consisted of counting the maximum number of impulses in a 1 s interval within the first 5 s of a recording using Autospike 32 software (Syntech, Germany). For the T184S mutant, which exhibited slower onset of response and lower impulse rates, the response was obtained by taking the average number of impulses per s between 10–15 s after stimulus onset. For screening the library of adenosine nucleotides, up to 10 stimulus chemicals were presented per fly; each chemical was tested on 3–6 sensilla on at least two flies. Responses were normalized to the response to 100 μM ATP (Sigma-Aldrich), which was presented after every third stimulus.

To validate agonist activity of selected chemicals, whole-cell transmembrane current responses were recorded in the whole-cell voltage-clamp configuration. HEK 293 cells heterologously expressing rat P2X2 receptor were held at a voltage of −60 mV and nucleotides were presented using a gravity-fed Rapid Perfusion System (RSC, Biologic) which was capable of exchanging solutions within 20 ms. Complete details of the methods and equipment employed for recording were as described by Wilkinson *et al.*^47^.

### Homology modeling and ligand docking

Homology modeling of rat P2X2 receptor was performed using Modeller (https://salilab.org/modeller/; Version 9.10)^68^ on the zebrafish P2X4 crystal structure in the ATP-bound open state (PDB ID 4DW1)^25^ as a template. Docking was performed using Schrödinger software (www.schrodinger.com; LigPrep, Epik, Glide-XP and Maestro: all from release 2015–1). Ligands were prepared (with LigPrep using the OPLS_2005 force field and Epik to generate ionization states at pH 7.0 ± 2.0), and docked into the ATP binding site of the molecular model using Glide-XP within a box of 22 Å. Poses were inspected visually and selected according to the lowest docking score values and maintenance of interactions known to be critical for agonist action. Images of docking poses were generated using UCSF Chimera (https://www.cgl.ucsf.edu/chimera/; Version 1.11)^69^.
